# Recombinant TBEV Protein E of the Siberian Subtype Is a Candidate Antigen in the ELISA Test System for Differential Diagnosis

**DOI:** 10.3390/diagnostics13203277

**Published:** 2023-10-23

**Authors:** Victoria Baryshnikova, Yuriy Turchenko, Ksenia Tuchynskaya, Ilmira Belyaletdinova, Alexander Butenko, Alena Dereventsova, Georgy Ignatiev, Ivan Kholodilov, Victor Larichev, Ekaterina Lyapeykova, Anastasiya Rogova, Armen Shakaryan, Anna Shishova, Anatoly Gmyl, Galina Karganova

**Affiliations:** 1 FSASI “Chumakov FSC R&D IBP RAS” (Institute of Poliomyelitis), Moscow 108819, Russiaturchenko.yu@mail.ru (Y.T.); belyaletdinova_i@mail.ru (I.B.); armen2@mail.ru (A.S.); a_shishova@list.ru (A.S.);; 2 D.I. Ivanovsky Institute of Virology Division of N.F. Gamaleya National Research Center of Epidemiology and Microbiology of the Ministry of Health of the Russian Federation, Moscow 123098, Russia; 3 Infectious Clinical Hospital No. 1 of the Moscow City Health Department, Moscow 125310, Russia; poleama@yandex.ru; 4 Department of Infectious Diseases in Children, Faculty of Pediatrics, Pirogov Russian National Research Medical University, Moscow 117997, Russia; 5 Institute of Translational Medicine and Biotechnology, Sechenov First Moscow State Medical University, Moscow 119991, Russia

**Keywords:** ELISA, TBEV, serodiagnosis of orthoflaviviruses, recombinant protein, protein E

## Abstract

The tick-borne encephalitis virus (TBEV) is one of the most common members of the *Orthoflavivirus* genus, which comprises the causative agents of severe diseases in humans and animals. Due to the expanding areas of orthoflavivirus infection, its differential diagnosis is highly demanded. Commercial test kits based on inactivated TBEV may not provide reliable differentiation between flaviviruses because of serological crossover in this genus. Application of recombinant domains (sE and dIII) of the TBEV Sukhar-strain protein E as antigens in an ELISA test system allowed us to identify a wide range of antibodies specific to different TBEV strains. We tested 53 sera from human patients with confirmed TBE diagnosis (the efficacy of our test system based on sE protein was 98%) and 56 sera from patients with other orthoflavivirus infections in which no positive ones were detected using our ELISA test system, thus being indicative of its 100% specificity. We also tested mouse and rabbit sera containing antibodies specific to 17 TBEV strains belonging to different subtypes; this assay exhibited high efficacy and differentiation ability in detecting antibodies against TBEV from other orthoflaviviruses such as Omsk hemorrhagic fever, Powassan, yellow fever, dengue, West Nile, Zika, and Japanese encephalitis viruses.

## 1. Introduction

The *Orthoflavivirus* genus comprises 53 species; 40 of them are pathogenic for humans or vertebrates, such as *Orthoflavivirus denguei* (dengue virus), *Orthoflavivirus flavi* (yellow fever virus—YFV), *Orthoflavivirus japonicum* (Japanese encephalitis virus—JEV), *Orthoflavivirus zikaense* (Zika virus), *Orthoflavivirus nilense* (West Nile virus—WNV), *Orthoflavivirus encephalitidis* (tick-borne encephalitis virus—TBEV), *Orthoflavivirus omskense* (Omsk hemorrhagic fever virus—OHFV), and *Orthoflavivirus powassanense* (Powassan virus—POWV) [1].

TBEV species can be divided into seven subtypes: European, Siberian, Far Eastern, Obskaya lineage, Himalayan, Baikalian-1, and Baikalian-2 ones [2]. TBEV infection can cause serious neurological disease and remains a real threat to public health. About ten thousand cases of tick-borne encephalitis (TBE) are annually registered in the world [3]. TBEV-endemic areas exhibit a high level of seropositive people [4,5]. The increase in internal and external tourism, as well as the presence of the combined foci of orthoflaviviruses in some regions, makes mixed infection possible, which, in turn, raises the question of differential diagnosis [6].

Orthoflavivirus infection is mainly diagnosed via serological tests [3,7]. A feature of the *Orthoflavivirus* genus is its antigenic cross-reactivity, which greatly complicates differential diagnosis [8,9,10]. Recently, only enzyme-linked immunosorbent assays (ELISA) have been used for routine diagnostics [11]. The application of inactivated TBEV as an immunogen for ELISA has become the standard, and the vast majority of commercially available test kits are based on it [12].

Protein E is the major antigen on the surface of TBEV, against which neutralizing antibodies are produced during infection. It consists of four domains: dI, dII, and dIII, which are exposed on the surface of the virus and compose the protein E ectodomain and membrane-associated dIV [13,14,15]. The structure of the protein E ectodomain was first studied using X-ray crystallography of the isolated soluble protein E (sE) [14]. Domains I and II contain most of the cross-reactive epitopes, while immunoglobulin-like domain III contains most of the virus-specific epitopes [16]. Domain III of protein E can be used as an antigen in differentiating test kits. Domains I and II can be used as antigens in test kits for detecting cross-reactive antibodies against orthoflaviviruses [17].

To increase the specificity of ELISA, various approaches have been described using recombinant proteins [18]. The previously described recombinant sE of TBEV and WNV, used in several studies as an antigen for ELISA, exhibited many cross-reactive epitopes; therefore, it had low specificity [17,19]. Several immunodiagnostics employing dIII as the basis for specific test kits for various orthoflaviviruses have been developed [20]. Recombinant dIII was successfully used to detect TBE-positive patients among sera containing antibodies specific to TBEV, dengue virus, and YFV [21]. Recombinant dIII from the strain Oshima 5 (the Far Eastern subtype of TBEV) showed a high sensitivity for TBE diagnostics [22]. The recombinant dIII of WNV was shown to differentiate anti-WNV antibodies in the sera of patients infected with WNV, JEV, and the St. Louis encephalitis virus [23]. The recombinant dIII of JEV was used to detect JEV-positive sera within a pool of sera containing antibodies to JEV, dengue virus, and chikungunya virus [24].

An alternative approach to diagnosing orthoflavivirus infection is to use the virus-like particles (VLPs) that are formed after the production of E and prM proteins, as well as ELISA based on the recombinant protein NS1 [25,26,27,28,29]. However, information is currently insufficient on the specificity and effectiveness of ELISA based on recombinant proteins for detecting antibodies to TBEV against a wide range of orthoflaviviruses.

This work describes new antigens for an ELISA system based on domain III or protein sE of the TBEV Sukhar strain of the Siberian subtype for performing differential identification of TBEV antibodies. A set of sera from patients with a confirmed diagnosis of TBE and other orthoflavivirus infections used in this test system showed the differential diagnostic potential of these recombinant proteins. A set of sera from mice with natural infections, as well as rabbit and mice hyperimmune sera infected with a wide range of TBEV strains belonging to several subtypes, was used in experiments to show that the test system detects a wide range of anti-TBEV antibodies.

## 2. Materials and Methods

### 2.1. Cells and Viruses

The porcine embryo kidney (PEK) cell line was maintained at 37 °C in medium 199 with Hanks’ balanced salt solution and Earle’s balanced salt solution (2:1, *v*/*v*, FSASI Chumakov FSC R&D IBP RAS, Moscow, Russia) supplemented with 5% fetal bovine serum (FBS, Invitrogen, Carlsbad, CA, USA).

**Figure 1:**
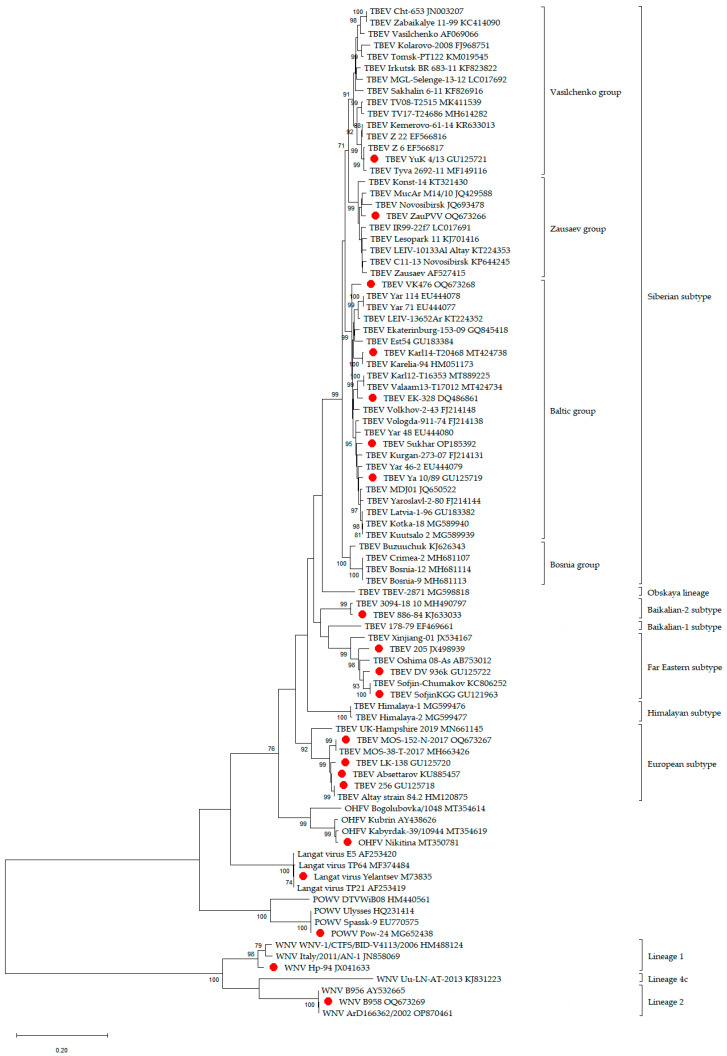
Phylogenetic tree of the *Orthoflavivirus* genus. Phylogenetic trees were constructed using protein E fragments (1097 bp) in MEGA X using the maximum likelihood method (1000 bootstrap replications). Bootstrap values (>70%) are shown at the branches. GenBank accession numbers are listed for each strain. Strains used in this work are marked with red circles.

Several TBEV strains belonging to the Far Eastern, European, Siberian, and Baikalian 1 and 2 TBEV subtypes, along with some other orthoflaviviruses, were used for the virus challenge and neutralization assay (Table 1, Figure 1). RNA sequences of TBEV strains from all main subtypes, along with strains of WNV, OHFV, POWV, and *Orthoflavivirus langatense* (Langat virus), were used in phylogenetic analysis. The nucleotide sequences of the genome-coding region of protein E (1097 bp) were aligned using ClustalW. Phylogenetic analysis was conducted using the maximum likelihood method and the Tamura–Nei model [30] in MEGA X with 1000 bootstrap replications [31].

### 2.2. Animals

Susceptible inbred BALB/c mice and outbred ICR mice (State Institution Scientific Center of Biotechnology, branch Stolbovaya, Stolbovaya settlement, Moscow Region, Russia) were used in this study. The animals were kept and treated in accordance with the international recommendations for the treatment of laboratory animals (CIOMS recommendations, 1985, Directive 2010/63/EU, and Appendix A to the European Convention ETS No. 123). The bioethics committee of FSASI Chumakov FSC R&D IBP RAS (protocol No. 17 of 1 September 2016) approved all the experimental procedures performed using animals.

### 2.3. Production of Recombinant Proteins sE and dIII

Recombinant domains of the protein E (sE and dIII) of the Siberian TBEV subtype strain Sukhar were used as antigens in the ELISA test system [33].

TBEV (Sukhar strain) RNA was extracted using TRIzol reagent (Thermo Fisher Scientific, Waltham, MA, USA). The cDNA was obtained using an MMLV RT kit (Evrogen, Moscow, Russia) following the manufacturer’s instructions. Target genes were cloned with Q5 polymerase (New England Biolabs, Ipswich, MA, USA), digested with corresponding restriction enzymes (Thermo Fisher Scientific, Waltham, MA, USA), and ligated into pQE vectors (Table 2). *E. coli* cells (strain JM109) were transiently transformed with the obtained plasmids. His-tagged recombinant viral antigens were purified on a Ni–NTA column (Bio-Rad, Hercules, CA, USA). After purification, the buffer was exchanged for a storage 1× PBS buffer (FSASI Chumakov FSC R&D IBP RAS, Moscow, Russia) using HiTrap desalting columns (Cytiva, Marlborough, MA, USA).

### 2.4. Collection of Mouse and Rabbit Sera and Ascitic Fluid

Mouse sera from natural infections were collected from three mice seven days post-subcutaneous TBEV inoculation and pooled. Virus strains are described in Table 1 and in the results section for each individual experiment. Mouse blood samples were obtained through decapitation; serum was separated from the clots by centrifugation and stored in aliquots at −20 °C.

Outbred mice (ICR) weighing 20–22 g were injected with 0.5 mL of a mixture of the virus and Freund’s complete adjuvant (1:1) (BD, Franklin Lakes, NJ, USA) under the skin at the withers (three times with a 7-day interval between injections) in order to obtain hyperimmune serum. Then, after 7 days, the virus without adjuvant was injected intraperitoneally (i/p). One day later, TG-180 sarcoma cells were injected i/p. After another 10–14 days, the mice were euthanized via decapitation. Blood and accumulated ascitic fluid were collected and centrifuged at 1500 rpm in an R6 centrifuge (Biosan). The supernatant was aliquoted and stored at −20 °C until use. All mouse sera used in the experiments were tested using a neutralization test against the relevant virus.

### 2.5. Human Sera

Six sets of human sera obtained from different sources were used in the work.

The first three sets were obtained from patients of Infectious Diseases Clinical Hospital No. 1 in Moscow, Russia. The first set was used for retrospective analysis over 15 years (Table 3). Upon admission to the hospital, all patients signed a form consenting to the use of their sera. Clinical diagnoses were confirmed via clinical symptoms and serological testing with a set of reagents for differential determination of IgM antibodies to the Zika, dengue, West Nile, and chikungunya viruses in human blood serum using “ELISA-IgM Zika, Dengue, ZN, Chik” (Branch MEDGAMAL of the Gamaleya National Research Center for Epidemiology and Microbiology of the Russian Ministry of Health, Moscow, Russia).

The second set of sera was obtained for retrospective analysis over 20 years. Twenty-six patients from the Vologda Region of Russia signed a form consenting to the use of their sera. All sera from clinical diagnoses were confirmed via clinical symptoms and serological testing with the VectoTBEV-IgG kit (Vector-Best, Novosibirsk, Russia) or the VectoTBEV-IgM kit (Vector-Best, Novosibirsk, Russia).

The third set of sera was obtained from patients admitted to the hospital in the period between 2017 and 2023. All patients provided voluntary informed consent to the use of sera. All patients were diagnosed with tick-borne encephalitis based on clinical presentation and serodiagnosis (Table 3).

The fourth set of human sera containing antibodies against various orthoflaviviruses was previously described in [34]. The sera were obtained from a double-blind clinical study in which participants gave voluntary, informed consent to the use of sera. All sera were tested for the presence of antibodies to ZIKV, ChikV, DENV, and YFV using a commercial ELISA kit (BioScreen-Chikungunya (IgG) and BioScreen-Dengue (IgG), Bioservice, Russia), an Anti-Zika Virus ELISA (IgG) kit (Euroimmun AG, Lübeck, Germany), and a Qualitative Human Yellow Fever Virus Antibody IgG (YFV-IgG) ELISA Kit (MyBioSource Inc., San Diego, CA, USA) (this ELISA Kit is only for research use).

**Figure 2:**
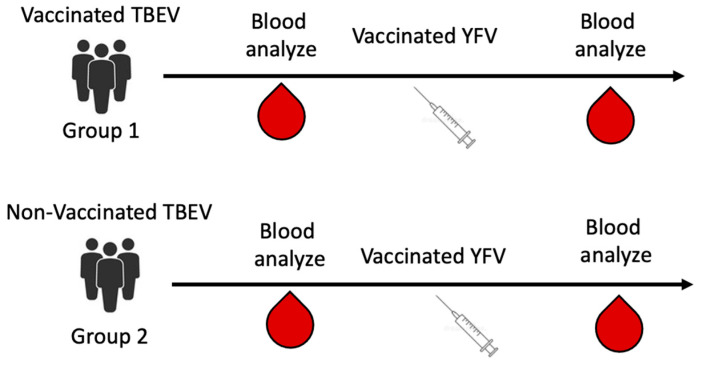
Scheme for obtaining blood for analysis in the clinical comparative study of the YFV vaccine.

The fifth set of sera from TBEV-vaccinated and non-vaccinated patients before and after YFV vaccination was previously obtained in a clinical comparative study of YFV vaccines (protocol JLH-III-01/20). All participants in the comparative study gave voluntary, informed consent to the use of sera. Sera of people vaccinated with the SinSaVac vaccine (LLC “Smartbiotech”, Moscow, Russia) were used. The scheme for obtaining the sera is shown in Figure 2. Previously vaccinated study participants received the EnceVir vaccine (Microgen, Moscow, Russia). The presence of anti-TBEV antibodies in the sera of vaccinated people was confirmed using an ELISA VectoTBE-IgG kit (Vector-Best, Novosibirsk, Russia).

The sixth set of sera was obtained in a healthy population from the Moscow Region of Russia, provided by the Center for Hygiene and Epidemiology in the Moscow Region. Patients signed an informed consent for the use of serum upon donation. It tested positive for anti-TBEV IgG antibodies using an ELISA VectoTBE-IgG kit (Vector-Best, Novosibirsk, Russia).

### 2.6. ELISA

Recombinant proteins (sE and dIII) or the negative internal control were placed into wells (12 ng per well) and incubated at 4 °C overnight. PEK or *E. coli* cell lysates or lysates of *E. coli* cells transformed with the pQE family plasmid, which had undergone the same isolation procedures as the recombinant proteins, were used as internal negative controls. Non-specific binding sites were blocked for 1 h at 37 °C with 4% skim milk in PBST (phosphate-buffered saline (FSASI Chumakov FSC R&D IBP RAS) plus 0.05% Tween 20 (Sigma, St. Louis, MO, USA)). The serum was diluted (1:100) and added to the plates for 1 h at 37 °C. After washing, the plates were incubated with the secondary antibody conjugated with horseradish peroxidase (Abcam, Boston, MA, USA) for 1 h at 37 °C. Substrate solution TMB (Sigma, St. Louis, MO, USA) was added to each well and incubated for 30 min at room temperature in the dark. Then, the reaction was stopped with 2 M of an H_2_SO_4_ solution (Lenreactiv, St. Petersburg, Russia). Results were detected through absorbance measurement at 450 nm (Multiscan, Thermo Fisher Scientific, Waltham, MA, USA). The serum was indicated as positive if the optical density of the sample was twice as high as that of the negative internal controls. We used titrated positive serum for the calibration curve to calculate the titer of the determined serum (an example is shown in Figure S1).

The VectoTBEV-IgG ELISA kit (Vector-Best, Novosibirsk, Russia) was used according to the manufacturer’s instructions.

### 2.7. The 50% Plaque Reduction Neutralization Test (PRNT_50_)

PRNT_50_ was performed on PEK cell monolayers in 24-well plates according to the procedure described earlier [35]. Briefly, dilutions of tested sera starting from 1:10 were incubated in medium 199 supplemented with 2% bovine FBS (Gibco, Waltham, MA, USA) in the presence of virus samples (1:1, *v*/*v*) at a concentration of 30–40 plaque formation units (PFUs) per well for 1 h at 37 °C. The virus–serum mixture (100 μL) was added to the PEK cells in the 24-well plates, and they were incubated for 1 h. Then, the wells were coated with 1.26% methylcellulose (Sigma, St. Louis, MO, USA) in medium 199 supplemented with Hanks’ and Earle’s salts (2:1, *v*/*v*) and 2% FBS (Gibco, Waltham, MA, USA) and left to incubate for 6 days at 37 °C in a CO_2_ incubator. Fixation and staining were performed with 96% ethanol and 0.4% gentian violet dye. Every experiment included controls, i.e., negative and positive murine sera with known antibody titers. The neutralization antibody (NAb) titers were calculated according to the modified Reed and Muench method [36].

## 3. Results

In this work, we used two types of TBEV-strain Sukhar-recombinant proteins as antigens for ELISA: dIII and sE. Different sets of human, mouse, and rabbit sera were used to determine the detection limit, efficacy, and specificity of our ELISA.

### 3.1. Efficacy of the ELISA Based on Recombinant TBEV Proteins

In this part of the work, in order to assess the efficacy and detection limit of the obtained recombinant antigens, we examined sera from patients with clinically and laboratory-confirmed TBE diagnoses from different regions of Russia taken at different stages of infection from three sets (# 1, 2, and 3, described in Materials and Methods) (Table 3) using ELISA.

Six out of seven sera from patients in the first set tested positive in the ELISA plates coated with both the sE and dIII domains (Table 4). One of the sera turned out to be negative in our test system, but in the neutralization test, the titer against the TBEV strain MOS-152-N-2017 belonging to the European subtype was 2.01 log at the threshold of the ELISA sensitivity. The second set of sera was also used in the studied recombinant protein-based ELISA test system. All the sera were positive for the ELISA coated with sE protein (Table 4). A total of 11 out of 26 sera were positive for ELISA coated with dIII protein (Table 4). All the sera from the third set were positive in the recombinant protein-based ELISA test system based on sE (20/20); when using dIII recombinant protein, 18 out of 20 sera were also positive.

Summary: Our test system shows 98% and 66% efficacy (52/53 and 35/53) based on sE and dIII, respectively, recombinant proteins from the Sukhar strain for testing human sera.

### 3.2. Detection Limit of the ELISA based on Recombinant TBEV Proteins

In order to assess the detection limit of the studied test system, we used a serial fourfold dilution starting from 1:100 serum of humans with confirmed TBE diagnosis, which had a NAb titer obtained from PRNT_50_ against TBE strain DV-936 1:1000. This serum was positive at a dilution of 1:100 in the commercial ELISA kit, at a dilution of 1:800 in the ELISA test kit based on protein sE, and at a dilution of 1:100 in the system based on dIII.

### 3.3. Specificity of the ELISA Based on Recombinant TBEV Proteins

The specificity of TBE antibody detection in the developed ELISA test system was shown using a pool of human sera of Japanese encephalitis (JE), Zika, West Nile (WN), and dengue patients with clinically and laboratory-confirmed infections (the first set) and containing antibodies to one or several orthoflavi- or alphaviruses previously described in [34] (the fourth set). All the tested sera were negative for the dIII or sE recombinant protein-based ELISA (Table 5).

One serum from a patient with JE showed cross-reactivity and tested positive in both the sE- and dIII-based ELISAs. We know that the patient (serum JE) was vaccinated against TBEV over 30 years ago and repeatedly visited areas in which TBEV is endemic. This serum was also studied using a neutralization assay. The NAb titer in the serum of the patient with JE against TBEV strain MOS-152-N-2017 was 2.11 log, being 2.8 log against JEV.

### 3.4. Effect of the Vaccination against YFV on TBEV Antibody Detection

It was also verified that the detection of antibodies after TBEV vaccination in human sera is not affected by vaccination against yellow fever (Figure 1). The sera from the people vaccinated against TBEV before and after vaccination against YFV (strain 17D) (the fifth set of human sera) tested positive in the developed recombinant protein-based ELISA test kit, both based on sE and dIII. Sera from people who were not vaccinated against TBEV tested negative both before and after administration of the yellow fever vaccine. Therefore, vaccination against YFV does not affect the detection of antibodies after vaccination against TBEV in the ELISA test kit based on recombinant sE and dIII.

### 3.5. Cross-Reactivity of the Studied Protein Used in ELISA with WNV-Positive Sera

Serological cross-reaction between antibodies to TBEV and WNV is one of the major problems in orthoflavivirus infection diagnostics. Ten serum samples from healthy humans from the Moscow region of Russia (the sixth set) were used. All the sera tested positive for anti-TBEV IgG antibodies using the commercial ELISA kit (VectoTBE-IgG) and were tested using a neutralization test with both the TBEV strain MOS-152-N-2017 and the WNV strain Hp-94 (Table 6).

**Figure 3:**
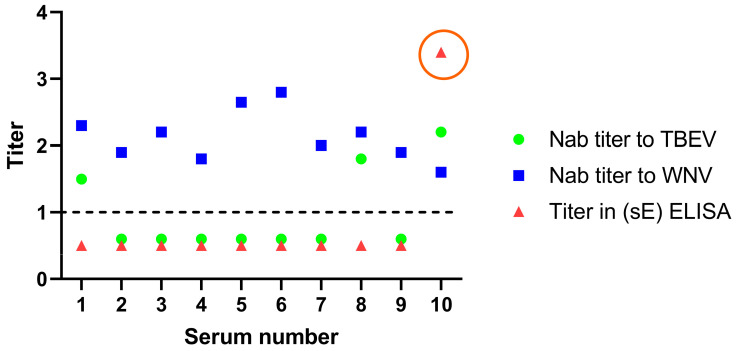
Comparison of the titer in the neutralization reaction and ELISA. The dashed line marks the threshold of the neutralization test and ELISA.

The NAb titer against TBEV was higher than that against WNV in serum #10 only (Table 6). All the sera were then analyzed using our ELISA test system. When sE recombinant proteins were used, all sera were negative except serum #10. The resulting data show the high specificity of our system since only serum #10 was positive in ELISA (Figure 3).

### 3.6. Specificity of the ELISA of the Hyperimmune Ascitic Fluids against Different Orthoflaviviruses and a Wide Range of TBEV Strains

Rabbit and mouse hyperimmune ascitic fluids of ICR mice against different TBEV strains and POWV, OHFV, Langat virus, and WNV were used. The resulting samples were tested in PRNT_50_ against the corresponding virus. The NAb titers of all the studied sera against the corresponding viruses in PRNT_50_ were >2 log. Sera with antibodies to POWV, OHFV, Langat virus, and WNV tested negative in our test system. Hyperimmune mouse and rabbit sera containing anti-TBEV antibodies to all the studied TBEV strains belonging to three main TBEV subtypes tested positive in ELISA based on sE and dIII recombinant proteins (Table 7).

In summary, we tested human and mouse sera containing antibodies to different orthoflaviviruses such as YFV, JEV, Zika virus, WNV, dengue virus, POWV, OHFV, and Langat virus; our ELISA test system based both on dIII and sE recombinant proteins showed 100% specificity.

### 3.7. Detection of Antibodies against a Wide Range of TBEV Strains from Mice with Natural Infection

Mouse sera from a natural infection and different TBEV strains were used to check the detection spectrum of the studied proteins (Table 8). Mice were infected subcutaneously with a set of TBEV strains related to different TBEV subtypes to obtain sera from a natural infection. Blood was taken from a total pool of three mice on day 7 seven after virus inoculation. All the sera were tested in PRNT_50_ against the strain of the corresponding subtype (Table 8).

Low NAb titers were obtained in the pools of mouse sera 7 days after virus injection, and 3/8 and 4/8 of the tested sera were negative to IgG antibodies in ELISA coated with sE and dIII recombinant proteins, respectively. Therefore, we decided to check if the tested sera contained IgM antibodies. Most of the sera from natural infection (7/8) tested positive against IgM antibodies in the ELISA with the dIII protein, and half of the pools (4/8) tested positive against IgM antibodies in the ELISA (Table 8).

## 4. Discussion

Serological research methods are an important component in the study of orthoflavivirus diseases [37]. ELISA, as one of these methods, can be used both in clinical diagnostics and in serological screening of populations living in regions where TBE is endemic [3] and is widely employed due to its simplicity, speed, and lack of special laboratory equipment requirements. The problems concerning insufficient diagnostic specificity when using ELISA test kits based on inactivated orthoflavivirus virions are widely known [38]. The use of recombinant proteins as antigens has been the main trend in developing modern test kits for TBEV and other orthoflaviviruses.

In this study, we used recombinant proteins sE and dIII of the Sukhar strain of the Siberian subtype of TBEV as antigens for designing a specific ELISA test system. dIII was chosen based on previous works where it was successfully used for specific serological diagnoses of different orthoflavivirus infections [18,20,21,22,23,24]. sE was used to investigate a possible increase in sensitivity without loss of specificity, although dI and dII, which are also present in the orthoflavivirus protein sE, are known to carry cross-reactive epitopes [16]. Nevertheless, our results show that sE captures a larger spectrum of antibodies than dIII alone and exhibits similar specificity. However, when detecting IgM antibodies during natural infection in the mouse sera on day 7 after virus injection, protein dIII was more effective.

Specific diagnosis of orthoflavivirus diseases is important for both co-circulating viruses and geographically and phylogenetically distant viruses under the conditions of active and developing tourism. Therefore, sE and dIII were tested against sera-containing antibodies to various orthoflaviviruses close to or distantly related to TBEV (Figure 1). The recombinant antigens showed good efficacy and specificity when tested with serum containing antibodies to individual orthoflaviviruses (TBE, Zika, WN, dengue, and YF) or mixed orthoflavivirus infections (Table 4 and Table 5).

We observed good specificity when testing sera that had shown inconsistent results in neutralization tests against TBEV and WNV (Table 6, Figure 3). This confirms that the studied recombinant proteins can be used as antigens with ELISA as a specific TBE diagnostic tool, which is especially important in areas that exhibit co-circulation of TBEV and WNV [3,39,40,41,42].

Moreover, the ELISA test system based on sE or dIII recombinant protein was effective when tested with the sera obtained from the patients from the different regions of Russia at the different stages of the natural TBE infection and at a long time after it (from 4 days to more than a year) (Table 3 and Table 4). In this work, we tested 109 sera of human patients with different orthoflavivirus infections. A total of 53 human sera were obtained from people with natural TBEV infection, of which 52 tested positive in our ELISA systems based on sE recombinant protein and 35 were positive with dIII recombinant protein, corresponding to efficacies of 98% for sE and 66% for dIII (Table 4). Furthermore, we showed the detection limit of the system, which was comparable with that of the commercial VectoTBEV-IgG kit, using one of the human serums in different dilutions. While the recombinant protein dIII exhibits high specificity, its efficacy as an antigen in the ELISA test kit is inadequate.

There was no positive result when testing 56 sera from patients with orthoflavivirus infection other than TBEV, which demonstrated 100% specificity of our ELISA test systems. A false-positive result in specificity testing proved interesting in a case of Japanese encephalitis imported to Moscow, described in a case report by doctors Petrova et al. [43]. A man infected with Japanese encephalitis in Thailand and already showing the acute stage of the disease in Moscow tested positive for IgG against JEV, TBEV, dengue virus, and WNV via ELISA. The diagnosis was made on the basis of a high anti-JEV antibody titer and the clinical and epidemiological picture. In addition, this patient had been vaccinated against tick-borne encephalitis 30 years before and could have visited regions in connection with work where tick-borne encephalitis is endemic. This can be the reason why antibodies to TBEV have been detected in a neutralization assay and ELISA with sE and dIII antigens. However, testing of JEV-only sera is necessary to eliminate the possibility of cross-reaction.

For yellow fever vaccination, no effect was shown on the efficacy of detecting anti-TBEV antibodies in the serum of patients vaccinated against TBEV in the ELISA with dIII and sE antigens.

In addition to specificity, we have shown for the first time the high sensitivity of our recombinant antigens to sera-containing antibodies to a wide range of TBEV strains belonging to European, Siberian, Far Eastern, and Baikalian subtypes. The characterized strains isolated in different years, from different sources, and from different geographical regions were used. Despite the phylogenetic difference (Figure 1), anti-TBEV antibodies in mouse hyperimmune and rabbit sera were unmistakably identified. As one can see from the OD values obtained in our experiments using mouse, rabbit, and human sera, the application of different proteins highlights the differences in antibody responses across species and within species, which can affect the diagnosis of difficult cases (Figure S2). Moreover, both hyperimmune sera, which have increased specificity, and the sera of mice on day 7 post-infection were investigated, thus illustrating the picture of a natural infection. Therefore, the spectrum of antibodies in the studied sera varies. Nevertheless, detection of IgG antibodies in mouse sera from natural infection proved problematic due to the early phase of infection, but repeating the test for IgM antibody detection corrected most of the false-negative results. We attribute this to the fact that the sera were taken quite early, at the first manifestations of the disease, so the IgG response was not yet fully formed.

As a result, we propose the recombinant protein sE of the Sukhar strain of TBEV as a platform for a future-specific and efficient ELISA test system.

## Figures and Tables

**Table 1:** Strains of TBEV, OHFV, WNV, Langat, and Powassan viruses were used in the present work.

Strain	Region and Year of Isolation	Source of Isolation	GenBank No.
TBEV Far Eastern subtype			
SofjinKGG	Primorsky Krai, Russia, 1937	Brain of deceased TBE patient	GU121963
205	Khabarovsky Krai, Russia, 1973	*I. persulcatus*	GU121964
DV-936k	Primorsky Krai, Russia, 1975	*I. persulcatus*	GU125722
TBEV Siberian subtype			
Sukhar	Yaroslavl’ region, 2016	Brain of deceased TBE patient	OP185392
EK-328	Estonia, 1972	*I. persulcatus*	DQ486861
Kurg-59	Kurgan region, 1989	*I. persulcatus*	
ZauPVV	Before 2000	unknown	OQ673266
VK476	Vologodski Region, 1975	* I. ricinus *	OQ673268
Karl14-T20468	Republic of Karelia, 2014	*I. persulcatus*	MT424738
Yuk 4/13	Kemerovo Region, Russia, 1969	*I. persulcatus*	GU125721
Ya-10/89	Yaroslavl’ region, 1989	*I. persulcatus*	GU125719
TBEV European subtype			
256	Belarus, 1940	* I. ricinus *	AF091014
Absettarov	Leningrad Region, Russia, 1951	Blood of a TBE patient	KU885457
MOS-152-N-2017	Moscow, 2017	* I. ricinus *	OQ673267 MH663429.1
LK-138	Lithuania, 1972	* I. ricinus *	GU125720
TBEV Baikalian-1 subtype			
178–79	Irkutsk Region, Russia, 1979	*I. persulcatus*	EF469661
TBEV Baikalian-2 subtype			
886–84	Irkutsk Region, Russia, 1984	Brain of gray-sided vole	EF469662
Powassan virus			
Pow-24	Primorsky Krai, Russia, 1976	*I. persulcatus* ticks	MG652438
OHFV, subtype 1			
Nikitina	Omsk Region, Russia, 1948	Blood of a patient with OHF	GU290187
Langat virus			
TP-21 (Elantsev) *	Malaya, 1956	*Ixodes granulatus*	M73835
WNV, Lineage 1			
Hp-94	Astrakhan Region, 1963	*Hyalomma marginatum*	JX041633.1
WNV, Lineage 2			
B958	Unknown, before 1980	Unknown	OQ673269
JE			
Gagar	Unknown, before 1975	Unknown	

* Showed one amino acid difference from TP-21 described by Mandl et al., 1991 [32].

**Table 2:** Primers used for recombinant protein cloning.

Protein	Primers
sE	For.: GAGCTACCATGGTCACACATCTGGAGAACAGG Rev.: TAGCTCAGATCTCTTTTGGAACCACTGATGGC
dIII	For.: GAGCTAGAGCTCACAATGTGTGACAAAACGAAG Rev.: TAGCTCAAGCTTCTTTTGGAACCACTGATGGC

**Table 3:** Human sera from Infectious Diseases Clinical Hospital No. 1 in Moscow, Russia, from patients admitted to the hospital before 2015 (set#1) and during the period between 2017 and 2023 (set#3).

Serum Name	Diagnosis	Sex	Age	Probable Site of Infection	Day of Disease
TBE (set#1)					
TBE1	TBE	F	12	Krasnoyarsk	21
TBE2		M	36	Ufa	180
TBE3		F	44	Chita	>365
TBE4		M	47	Crimea	>180
TBE5		M	38	Chelyabinsk	28
TBE6		M	?	Vologda	6
TBEvac *		F	26	Moscow	
TBE (set#3)					
TBE7	TBE	M	2.5	unknown	unknown
TBE8		M	unknown	unknown	unknown
TBE9		M	unknown	unknown	unknown
TBE10		F	29	Altai region, Russia	7
TBE11		M	unknown	unknown	unknown
TBE12		M	50	Chelyabinsk Region, Russia	47
TBE13		M	42	Tver Region, Russia	5
TBE14		M	19	Novosibirsk Region, Russia	9
TBE15		M	36	Perm Region, Russia	26
TBE16		M	unknown	unknown	unknown
TBE17		M	45	Perm Region, Russia	13
TBE18		M	42	unknown	11
TBE19		M	42	unknown	14
TBE20		M	42	unknown	15
TBE21		M	unknown	Republic of Karelia, Russia	14
TBE22		F	50	Kostroma Region, Russia	22
TBE23		F	5	Republic of Khakassia, Russia	unknown
TBE24		M	52	unknown	27
TBE25		M	42	Tver Region, Russia	14
TBE26		M	19	Novosibirsk Region, Russia	16
	JE(set#1)				
JE **	JE	M	53	Thailand	18
	Zika(set#1)				
Zika1	Zika	F	35	Thailand	37
Zika2		M	?	Dominican	14
	WN(set#1)				
WN1	WN	F	32	Moscow	14
WN2		F	63	Volgograd	7
WN3		M	43	India	15
WN4		M	32	India	21
WN5		F	24	Volgograd	>180
WN6		M	?	Astrakhan	unknown
	Dengue(set#1)				
D1	dengue	F	56	Maldives	7
D2		M	45	Shri Lanka	6
D3		F	29	Maldives	7
D4		M	28	Vietnam	14
D5		F	40	Thailand	9
D6		M	?	Vietnam	8

* The patient was vaccinated twice with the Tick-E-Vac vaccine. ** The patient was vaccinated against TBEV 30 years ago and repeatedly visited endemic areas of TBE.

**Table 4:** Analysis of the human sera containing TBEV antibodies in the recombinant protein-based ELISA test system.

Set #	Number of Sera, *n*	ELISA (sE)	ELISA (dIII)
1	7	6/7	6/7
2	26	26/26	11/26
3	20	20/20	18/20

**Table 5:** Analysis of the human serum containing antibodies to closely related orthoflaviviruses in the recombinant protein-based ELISA test system. JE = Japanese encephalitis virus; YF = yellow fever virus; Zika = Zika virus; Chick = chikungunya virus (alphavirus).

Set #	Diagnosis	Number of Sera, *n*	ELISA (sE)	ELISA (dIII)
1	JE	1	1/1	1/1
	Zika	2	0/2	0/2
	WNV	6	0/6	0/1
	Dengue	6	0/6	0/6
4	YF	5	0/5	0/5
	YF + Zika	1	0/1	0/1
	Chick +YF	5	0/5	0/5
	Chick + Zika	3	0/3	0/3
	Chick	3	0/3	0/3

**Table 6:** The neutralization antibody titer (NAb) in human sera was PRNT_50_ against TBEV and WNV.

Serum #	NAb Titer against TBEV, log(NAb)	NAb Titer against WNV, log(NAb)
1	1.5 *	2.3
2	<1 **	1.9
3	<1	>2.2
4	<1	1.8
5	<1	2.7
6	<1	>2.8
7	<1	2
8	1.8	2.2
9	<1	1.9
10	>2.2	1.6

* The scatter of values for PRNT50 with control serum for TBEV is within SD = 0.18 (*n* = 10); ** The value falls below the sensitivity threshold of the method.

**Table 7:** NAb titers and the results of the ELISA in mouse and rabbit hyperimmune ascitic fluids to different orthoflaviviruses.

Hyperimmune Serum	Virus, Strain	NAb Titers to Corresponding Virus, log(NAb)	ELISA (sE)	ELISA (dIII)
Mouse	Powassan virus, Pow-24	>3.4 *	−	−
	OHFV, Nikitina	2.2	−	−
	Langat virus, TP-21 (Elantsev)	2.5 ^1^	−	−
	WNV, B958	2.3	−	−
	TBEV, Absettarov	2.95	+	+
	TBEV, ZauPVV	1.9 ^2^	+	+
	TBEV, VK476	2.94	+	+
	TBEV, MOS-152-N-2017	1.9	+	+
	TBEV, Karl14-T20468	2	+	+
Rabbit	TBEV, 256	n/t	+	+
	TBEV, Yar-82	n/t	+	+
	TBEV, Kurg-59	n/t	+	+

* The scatter of values for PRNT50 with control serum for TBEV is within SD = 0.18 (*n* = 10); ^1^ NAb titer was tested in PRNT50 with OHFV; ^2^ NAb titer was tested in PRNT50 with strain MOS-152-N-2017; n/t = not tested.

**Table 8:** Results in the ELISA test system for mouse sera after natural infection with different strains of TBEV.

Serum	Subtype	Strain	NAb Titer against TBEV	ELISA of IgG (sE)	ELISA of IgM (sE)	ELISA of IgM (dIII)
Mouse	European	Absettarov	n/t ^1^	+	+	+
	European	LK-138	1.25 * ^1^	−	−	+
	Siberian	Yuk 4/13	1.07 ^2^	+	+	+
	Far Eastern	SofjinKGG	1.17 ^3^	+	−	−
	Far Eastern	205	1.24 ^3^	+	+	+
	Far Eastern	DV 936k	n/t ^3^	+	+	+
	Baikalian-1	178–79	1.48 ^2^	−	−	+
	Baikalian-2	886–84	1.45 ^2^	−	−	+

* The scatter of values for PRNT50 with control serum for TBEV is within SD = 0.18 (*n* = 10); ^1^ NAb titer was tested in PRNT_50_ with LK-138; ^2^ NAb titer was tested in PRNT_50_ with EK-328; ^3^ NAb titer was tested in PRNT_50_ with SofjinKGG; n/t = not tested.

## Data Availability

Not applicable.

## Supplementary Materials

The following supporting information can be downloaded at: https://www.mdpi.com/article/10.3390/diagnostics13203277/s1, Figure S1: An example of a calibration curve to calculate the titer of the determined serum of the patient vaccinated against TBEV by the ‘Tick-E-Vac’ vaccine; Figure S2: OD values of positive for the presence of TBEV antibodies in serum from people, mice, and rabbits obtained from different ELISAs, showing the difference in the antibody response to different proteins.

## Appendix A

Available online: https://www.coe.int/t/e/legal_affairs/legal_co-operation/biological_safety_and_use_of_animals/laboratory_animals/2006/Cons123(2006)3AppendixA_en.pdf (accessed on 15 June 2006).
