# Supplementary material for: Recombinant TBEV Protein E of the Siberian Subtype Is a Candidate Antigen in the ELISA Test System for Differential Diagnosis
Source: Diagnostics (Basel). 2023 Oct 23;13(20):3277. doi: 10.3390/diagnostics13203277 (PMC10606673; doi:10.3390/diagnostics13203277)
Supplement: Supplementary file 1 [file diagnostics-13-03277-s001.zip › diagnostics-2275459-supplementary.pdf]

Supplementary Information

Recombinant TBEV protein E of the Siberian subtype is a candidate antigen in the ELISA test system for differential diagnostics

Victoria Baryshnikova [1,†], Yuriy Turchenko [1,†], Ksenia Tuchynskaya [1,†], Ilmira Belyaletdinova [1], Alexander Butenko [2], Alena Dereventsova [1], Georgy Ignatiev [1], Ivan Kholodilov [1], Victor Larichev [2], Ekaterina Lyapeykova [3], Anastasiya Rogova [1], Armen Shakaryan [1,4], Anna Shishova [1,5], Anatoly Gmyl [1,‡] and Galina Karganova [1,5,*]

1 FSASI "Chumakov FSC R&D IBP RAS" (Institute of Poliomyelitis), Moscow 108819, Russia
2 D.I. Ivanovsky Institute of Virology Division of N.F. Gamaleya National Research Center of Epidemiology and Microbiology of the Ministry of Health of the Russian Federation, Moscow 123098, Russia
3 Infectious Clinical Hospital No. 1 of the Moscow City Health Department, Moscow 125310, Russia
4 Pirogov Russian National Research Medical University, Department of Infectious Diseases in Children, Fac-ulty of Pediatrics, Moscow 117997, Russia
5 Institute of Translational Medicine and Biotechnology, Sechenov First Moscow State Medical University, Mos-cow 119991, Russia
* Correspondence: karganova@bk.ru
† These authors contributed equally to this work.
‡ Deceased

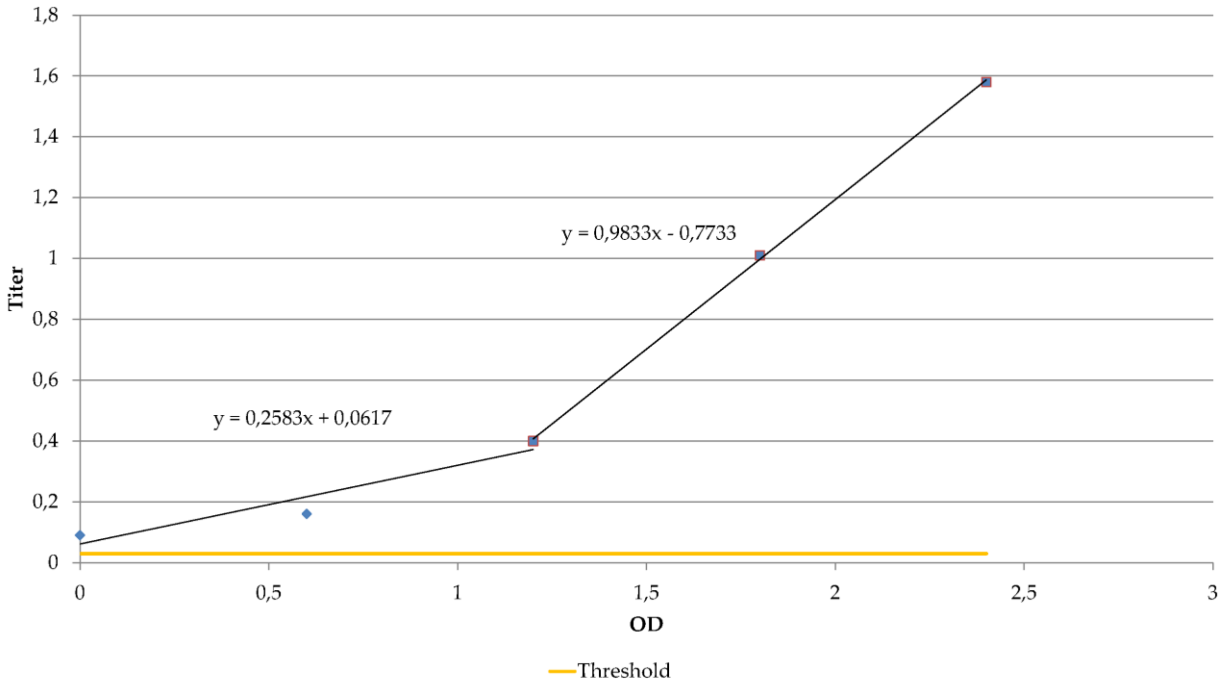

Figure S1. An example of a calibration curve to calculate the titer of the determined serum of the patient vaccinated against TBEV by ‘Tick-E-Vac’ vaccine.

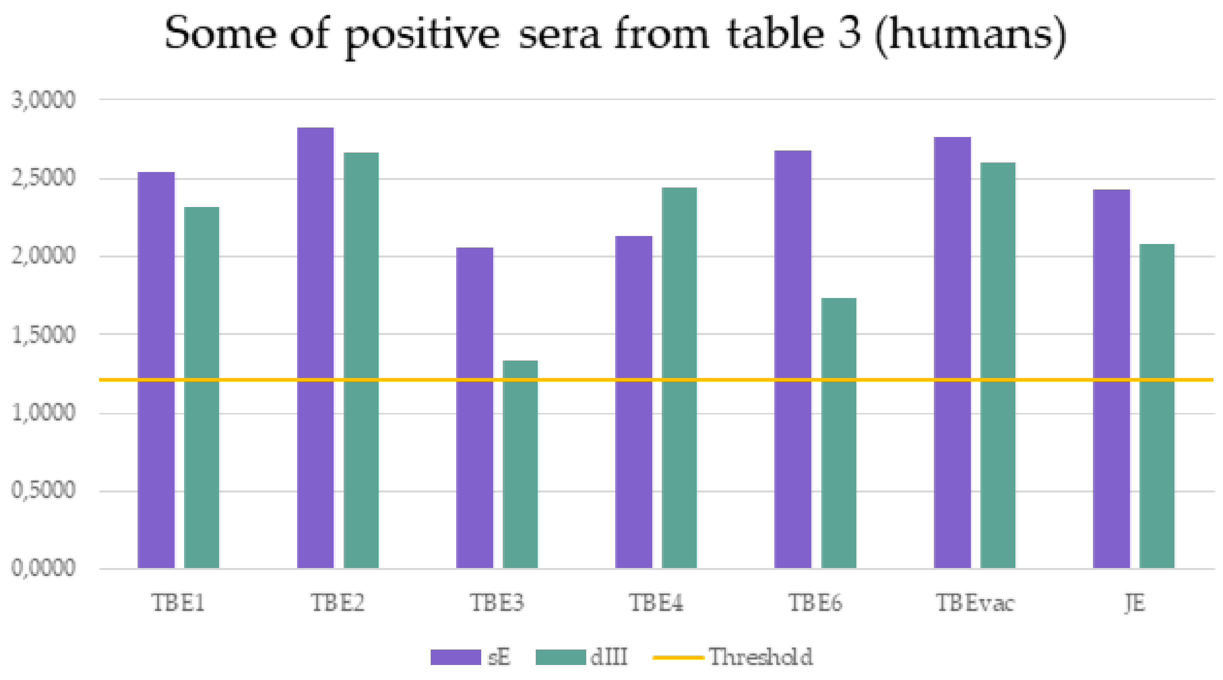

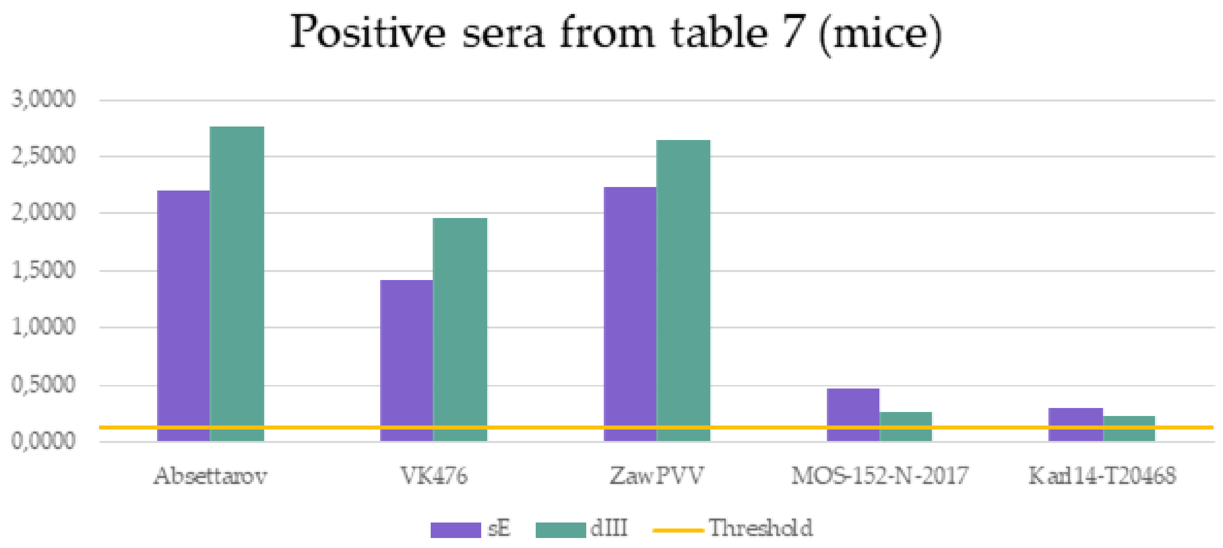

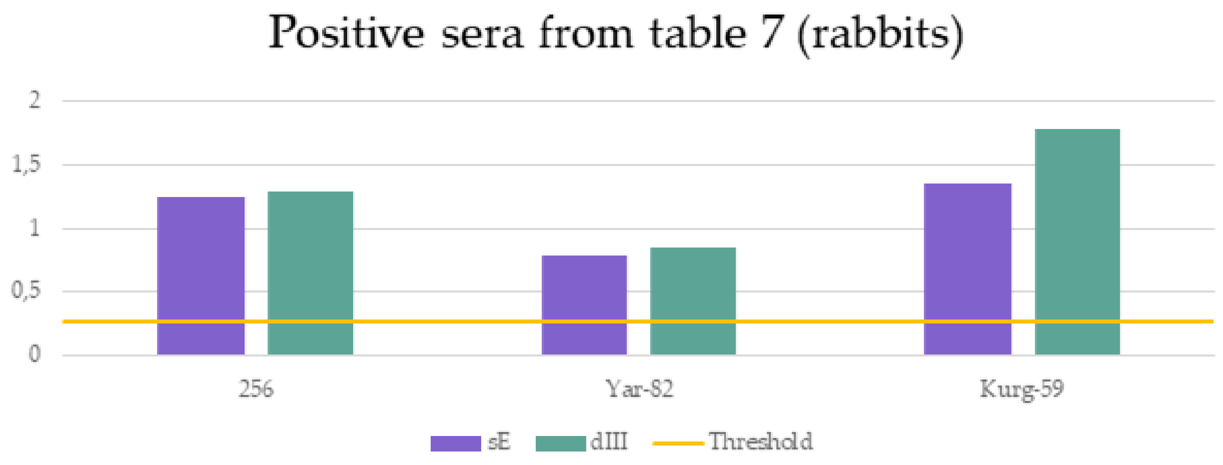

Figure S2. OD values of positive to presence of TBEV antibodies serum from people, mice and rabbits obtained from different ELISA, showing the difference in the antibody response to different proteins
